# Comparison of Internet and Telephone Interventions for Weight Loss Among Cancer Survivors: Randomized Controlled Trial and Feasibility Study

**DOI:** 10.2196/cancer.7166

**Published:** 2017-09-27

**Authors:** Matthew Cox, Karen Basen-Engquist, Cindy L Carmack, Janice Blalock, Yisheng Li, James Murray, Louis Pisters, Miguel Rodriguez-Bigas, Jaejoon Song, Emily Cox-Martin, Wendy Demark-Wahnefried

**Affiliations:** ^1^ Adult and Child Consortium for Health Outcomes Research and Delivery Science School of Medicine University of Colorado Aurora, CO United States; ^2^ Center for Energy Balance In Cancer Prevention and Survivorship Department of Behavioral Science MD Anderson Cancer Center, University of Texas Houston, TX United States; ^3^ Department of Palliative Care and Rehabilitation Medicine MD Anderson Cancer Center, University of Texas Houston, TX United States; ^4^ Tobacco Treatment Program Department of Behavioral MD Anderson Cancer Center, University of Texas Houston, TX United States; ^5^ Department of Biostatistics MD Anderson Cancer Center, University of Texas Houston, TX United States; ^6^ Department of Breast Medical Oncology MD Anderson Cancer Center, University of Texas Houston, TX United States; ^7^ Department of Urology MD Anderson Cancer Center, University of Texas Houston, TX United States; ^8^ Department of Surgical Oncology MD Anderson Cancer Center, University of Texas Houston, TX United States; ^9^ Division of Medical Oncology University of Colorado Aurora, CO United States; ^10^ Department of Nutrition Sciences University of Alabama Birmingham, AL United States

**Keywords:** weight loss intervention, cancer survivors, Internet, telephone

## Abstract

**Background:**

Weight loss interventions have been successfully delivered via several modalities, but recent research has focused on more disseminable and sustainable means such as telephone- or Internet-based platforms.

**Objective:**

The aim of this study was to compare an Internet-delivered weight loss intervention to a comparable telephone-delivered weight loss intervention.

**Methods:**

This randomized pilot study examined the effects of 6-month telephone- and Internet-delivered social cognitive theory–based weight loss interventions among 37 cancer survivors. Measures of body composition, physical activity, diet, and physical performance were the outcomes of interest.

**Results:**

Participants in the telephone intervention (n=13) showed greater decreases in waist circumference (–0.75 cm for telephone vs –0.09 cm for Internet, *P*=.03) than the Internet condition (n=24), and several other outcomes trended in the same direction. Measures of engagement (eg, number of telephone sessions completed and number of log-ins) suggest differences between groups which may account for the difference in outcomes.

**Conclusions:**

Cancer survivors in the telephone group evidenced better health outcomes than the Internet group. Group differences may be due to higher engagement in the telephone group. Incorporating a telephone-based component into existing weight loss programs for cancer survivors may help enhance the reach of the intervention while minimizing costs. More research is needed on how to combine Internet and telephone weight loss intervention components so as to maximize engagement and outcomes.

**Trial Registration:**

ClinicalTrials.gov NCT01311856; https://clinicaltrials.gov/ct2/show/NCT01311856 (Archived by WebCite at http://www.webcitation.org/6tKdklShY)

## Introduction

Weight gain, a common and worrisome side effect of certain cancer treatments such as chemotherapy [1-3], can persist after treatment and increases the risk for chronic diseases as well as cancer recurrence and second primaries [1,4-6]. Weight gain that occurs postdiagnosis may be associated with poorer disease-specific and overall survival [7,8]. For example, a recent meta-analysis of postdiagnosis weight gain in breast cancer survivors showed that a 5% weight gain was associated with a 12% increase in all-cause mortality, and a 10% weight gain was associate with a 23% increase in all-cause mortality [9]. Two meta-analyses in breast and prostate cancer survivors showed that postdiagnosis increases in body mass index (BMI) are significantly associated with greater recurrence as well as poorer disease-free and overall survival [10,11]. Given the physical, economic, and psychological burdens that cancer survivors face, recent intervention efforts to prevent recurrence and ameliorate symptoms in posttreatment cancer survivors have shown promise.

Diet and exercise interventions may facilitate weight management in survivors [12,13]. In order to increase the reach of weight loss interventions and decrease costs, distance-based approaches using communication technology, such as telephone counseling, are receiving more attention. A recent review of weight loss interventions for breast cancer survivors identified 3 randomized controlled trials (RCT) where at least 1 component of the intervention was delivered via telephone [14]. Authors noted that only 2 of these studies compared a telephone-delivered intervention to a non–telephone-delivered intervention, and only 1 of these 2 studies reported any statistical comparisons between intervention conditions. Although this study reported that the telephone intervention condition achieved significantly more weight loss than 2 other active control conditions, Reeves et al [14] rated the risk for bias of the results as high based on a checklist created from the Consolidated Standards of Reporting Trials (CONSORT) statement and the *Cochrane Handbook for Systematic Reviews of Interventions*.

Moreover, a recent systematic review of Web-, telephone-, and print-based interventions targeting weight management in cancer survivors found only 5 studies that targeted weight management and only 2 studies that found significant improvement in weight status [15]. All 5 interventions used telephone-based intervention methods, with 1 RCT showing that a telephone intervention was significantly effective in reducing BMI among 641 older, overweight or obese colon, breast, and prostate cancer survivors [16]. As minimal as such an approach appears, it still requires dedicated staff and resources. In order to reduce these costs, less expensive means to deliver interventions are sought.

Web-based delivery is one way to reduce cost and expand the reach of weight loss interventions. There are numerous review articles and meta-analyses examining weight loss or weight control interventions delivered via the Internet. Overall, reports suggest that half of the interventions were successful in promoting weight loss or weight maintenance; however, the interventions as well as the effects were heterogeneous, limiting the ability to identify critical components. Neve et al [17] identified 7 studies for inclusion in their meta-analysis of Web-based interventions for weight loss and weight loss maintenance in overweight and obese adults; however, only 4 of the Web-based interventions were deemed effective and included in the meta-analysis. Results showed no difference between the Web-based interventions and the control condition because of substantial heterogeneity in results. In a larger meta-analysis, Kodaman et al [18] examined 23 RCTs of Web-based weight loss interventions, finding a modest but significant effect for weight loss with the Web-based intervention as compared to the control condition (–0.68 kg). The authors also found significant heterogeneity in results, which were dependent on the other components included in the intervention.

In a systematic review of reviews, Tang et al [19] found 4 meta-analyses examining Internet-based interventions for weight loss. While the authors noted heterogeneity both within and across the meta-analyses, they observed that these interventions were consistently more effective than minimal contact interventions (eg, printed material) and that interventions using self-monitoring and feedback showed promise for improving weight loss as opposed to information-only interventions.

A consistent issue noted in several review articles was the lack of use of the Internet-based materials by participants. Norma et al [20] reviewed 41 studies comparing interventions using eHealth technology to control groups and suggested that studies with higher usage rates had improved outcomes, but the authors failed to note a critical number of log-ins to achieve these results. In a review of Web-based physical activity interventions, Vandelanotte et al [21] noted that interventions with 5 or more contacts had higher levels of reported physical activity. Arem and Irwin [22] observed a similar association between log-in rates and weight loss but noted that exceptions do occur, citing one study in particular that incentivized log-ins and still did not produce clinically significant weight loss [23].

In the reviews and meta-analyses examining weight loss interventions delivered via Internet, it was found that no studies examined the impact of Internet-based approaches among cancer survivors who tend to be older [24] and less likely to use the Internet and other forms of technology [25]. Moreover, these reviews largely compared telephone- or Web-based delivery modalities with those that were face-to-face or versus waitlist controls. No direct comparisons have been made, so claims about the comparative efficacy of telephone- versus Internet-delivered interventions cannot be made. Given that no previous study has directly compared a telephone- versus Web-based weight loss intervention in either the general population or among cancer survivors, we believe a pilot study is warranted in order to develop estimates of effect sizes for future studies.

Determining the modality that provides the largest reach with the most weight loss will help to identify the most effective intervention approach for weight loss. Our study attempts to bridge the gap in the literature by directly comparing a tailored telephone- versus Internet-delivered weight loss intervention among cancer survivors. Based on the current literature, we hypothesize that the telephone group will have greater weight loss and more improved health outcomes than the Internet group.

## Methods

### Ethical Approval

All procedures performed in studies involving human participants were in accordance with the ethical standards of the institutional or national research committee and with the 1964 Helsinki declaration and its later amendments or comparable ethical standards.

### Participants

Participants were 37 cancer survivors who had previously participated in a survey about health behavior change interventions and delivery modalities and indicated that they were willing to be contacted for participation in future studies. Participants had to have a diagnosis of either locoregional breast cancer (stages 0 to IIIA), colon cancer (stages I and II), endometrial cancer (stages I to IIIa), or prostate cancer (stages I and II) and no history of any other cancers. Participants were required to be at least 3 months postsurgery (if applicable), over the age of 18 years, have a BMI ≥25, have access to high-speed Internet and a telephone, and live in the Houston area. Survivors were excluded if they had a medical condition that prevented them from engaging in an unsupervised exercise program or low-fat diet high in fruits and vegetables. Informed consent was obtained from all individual participants included in the study. In-person assessments were completed at baseline and 6 months for all measures.

### Measures

#### Body Composition

Percent body fat was measured using the whole body Discovery A QDR x-ray bone densitometer (Hologic Inc) (daily quality control was performed using the phantom spine). Additionally, researchers weighed participants and measured their waist circumference at both time points. Height was measured at baseline and was used with weight to calculate a BMI (kg/m^2^) for each participant.

#### Diet

The online Automated Self-Administered 24-hour (ASA24) dietary recall was used to document participant food intake (riskfactor.cancer.gov/tools/instruments/asa24). Two assessments (1 for weekday and 1 for weekend day) were obtained and averaged. Results related to intakes of energy, saturated fat, fiber, and number of servings of fruits and vegetables were outcomes of interest.

#### Physical Activity

A 3-item modified version of the Godin Leisure-Time Physical Activity Questionnaire was used to measure participant usual leisure-time exercise habits. This questionnaire has been used extensively in research with cancer survivors. It is easy to administer and has good test-retest reliability (.81 for total score) and significant correlations with maximal oxygen consumption (VO_2_ max) [26]. For 1 week before the baseline and 6-month assessments, participants wore a GT1M accelerometer (Actigraph LLC) and recorded their exercise in a daily diary. Participants were asked to indicate what type of exercise they performed, duration of the exercise in minutes, and the effort level during the exercise. In terms of outcomes, the Godin was used to develop a total score of physical activity minutes as well as a measure of moderate/vigorous physical activity minutes. The accelerometer was used to measure the number of sedentary minutes and the percentage of the day that participants engaged in moderate/vigorous physical activity. Cut-points for sedentary minutes and minutes of moderate/vigorous physical activity were derived using the methods of Hall et al [27].

#### Physical Performance

For aerobic function, a 2-minute step-in-place protocol was used. The 2-minute step-in-place protocol assesses the number of steps within 2 minutes a participant can complete in place by raising their knees to a height halfway between the iliac crest and the middle of the patella. This test correlates moderately with other common measures of aerobic capacity and is low risk [28]. For lower body strength, a 30-second chair-stand test was used [29], in which the number of full stands in a 30-second period was recorded. We used a timed arm curl task to assess upper body strength and functionality [29]. Upper body function, including arm strength and endurance, is important in activities of daily living such as carrying groceries, lifting purses, etc. Timed arm flexion tasks simulate these activities. To assess agility and dynamic balance, an 8-foot up-and-go assessment was used. The test is a modification of the 3-meter timed up-and-go test [30] and can be administered in small spaces [29]. The 6-minute walk test was used as a measure of endurance. It has been validated in older adults against treadmill walking tests resulting in a correlation of .78 [31].

### Procedures

This study was approved by MD Anderson’s Institutional Review Board and was registered at ClinicalTrials.gov [NCT01311856]. Following the baseline assessment, participants were randomized at a ratio of 2:1 to either the Internet-based weight loss intervention or the telephone-based version, respectively. A 2:1 ratio was used because we hypothesized that outcomes in the Internet condition would be smaller. We used a form of adaptive randomization called minimization, which is similar to stratification in that participant characteristics are used to assign them to the treatment conditions [32,33]. All participants received resistance bands and pedometers. Participants in the telephone intervention received print materials about exercise and diet and telephone counseling calls (3 weekly, 2 semiweekly, 4 monthly; 15 to 30 minutes in length) and customized mailed progress reports every 6 weeks to encourage adherence to diet and exercise recommendations. Materials were based on the Reach Out to Enhance Wellness (RENEW) intervention [16]. Participants in the Internet-based intervention had access to the same content online by logging onto www.walkingspree.com/login/healthymoves with a personalized username and password. Participants in the Internet arm were also invited to participate in a discussion forum facilitated by intervention staff, had the opportunity to email questions directly to the intervention staff, and received customized progress reports every 6 weeks by email.

The goals for both groups were to do 15 minutes of strength exercise every other day, ≥30 minutes of walking or other moderate-intensity exercise on 5 or more days of the week, and consume a diet with 7 (for women) or 9 (for men) servings of fruits and vegetables per day and <7% of calories from saturated fat. Participants in both groups were also provided with caloric recommendations to facilitate a weight loss of 1 to 2 pounds per week (a loss of 5% body weight was used as a goal over the course of the 6-month study period) and fat gram/calorie counters or access to appropriate websites to monitor intake. Participants received 2 $25 gift cards as compensation; 1 after completing the baseline assessment and the other after completing the 6-month assessment.

### Analyses

Two-sample and paired *t* tests and Fisher exact tests with a 2-sided alpha of .05 were used to (1) compare the 2 intervention groups on a number of demographic variables; (2) compare the difference scores from baseline to 6 months on diet, physical activity, physical performance, and body composition between the 2 intervention groups; (3) assess within-group changes from baseline to 6 months on the aforementioned outcome variables; and (4) compare attrition rates between intervention groups for each outcome variable. We define attrition here as any participant who completed baseline measures but stopped participating at some point following baseline (eg, the participant dropped out of the study and no further data were collected). Additionally, Cohen *d* was calculated for within-group differences between baseline and 6 months.

## Results

**Figure 1:**
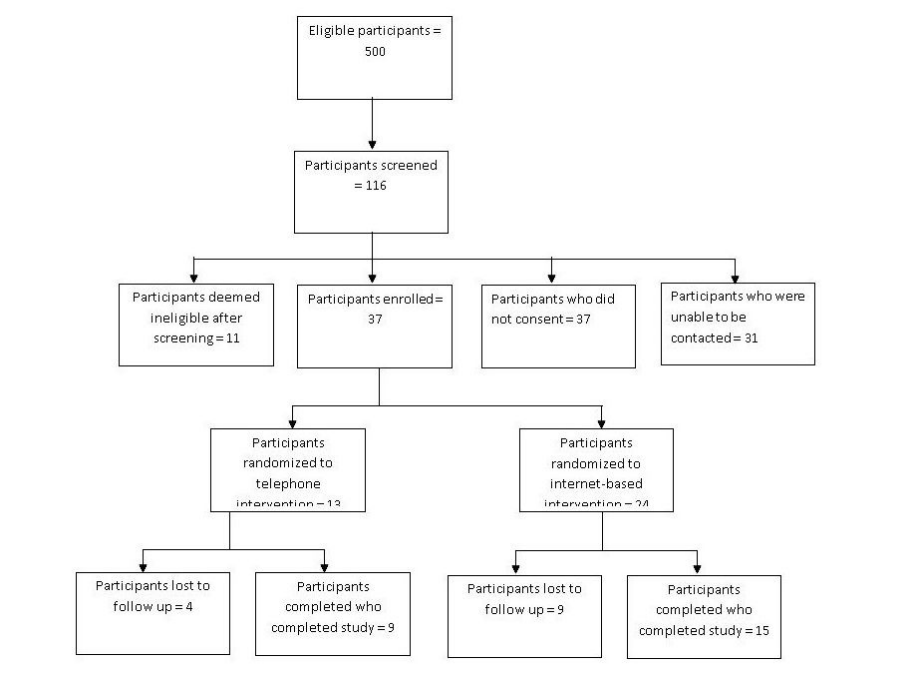
Consolidated Standards of Reporting Trials diagram.

Participants included 37 cancer survivors. A CONSORT diagram for recruitment and retention is presented in Figure 1. Baseline demographic information by intervention group is presented in Table 1. No significant differences were observed in any of these parameters. Despite the lack of statistical significance on these parameters, the distribution of ethnicities appears to be substantially different between the 2 intervention conditions, with the Web-based condition having substantially more white participants.

Attrition did not differ significantly between the 2 treatment groups. Participants who did not complete their 6-month assessment were only different in terms of their baseline percentage body fat, with those dropping out having a higher percentage of body fat than those who did not (noncompleters: mean 51.85 (SD 3.81) kg/m^2^; completers: mean 41.49 (SD 4.23) kg/m^2^, *P*=.002). This difference was only noted in the telephone-based intervention group. Additionally, potentially differential levels of engagement were observed between the 2 intervention groups. On average, participants completed 7.2 out of 9 telephone counseling sessions (80%) in the telephone-based group, while participants logged in 43.2 days out of a possible 160 days (27%) in the Internet-based group. Another more comparable measure of engagement was the tailored weekly online survey that participants completed. The telephone group had a higher percentage of completion than the Internet group (60% vs 42%) (see Multimedia Appendix 1 for a table of comparisons for completers vs noncompleters on baseline outcomes).

Results of the *t* tests comparing within intervention group differences between baseline and 6 months are presented in Table 2. Significant changes over time for the telephone group included decreases in weight (*D*=0.81, *P*=.04), waist circumference (*D*=1.01, *P*=.02), and 8-foot up-and-go times (*D*=0.84, *P*=.04), and while a decrease in BMI was substantial, it was not statistically significant (*D*=0.75, *P*=.06). The Internet-based group showed increases over time in body fat percentage (*D*=0.98, *P*=.004) but improvement in 2 performance tasks: the 30-second bicep curl (*D*=0.71, *P*=.02) and the 30-second sit-to-stand (*D*=0.73, *P*=.02).

**Figure 2:**
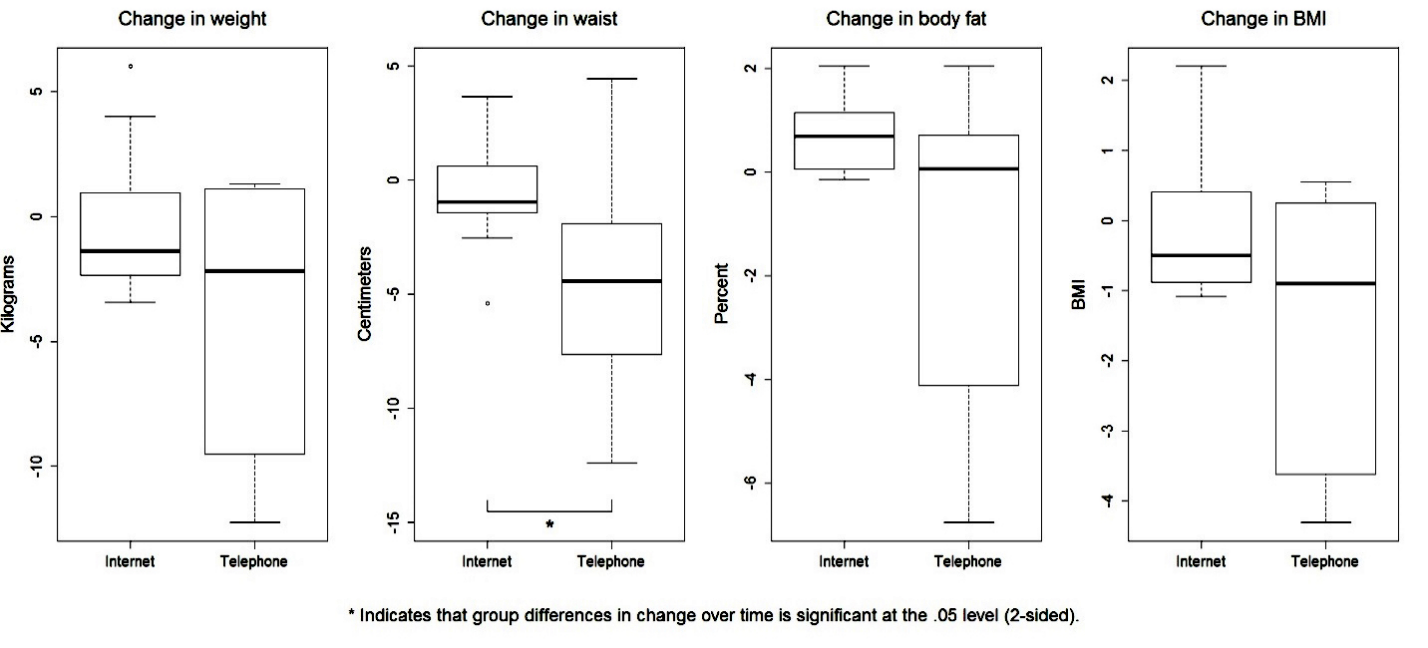
Boxplots for change in body composition by treatment group from baseline to 6-month follow-up.

**Figure 3:**
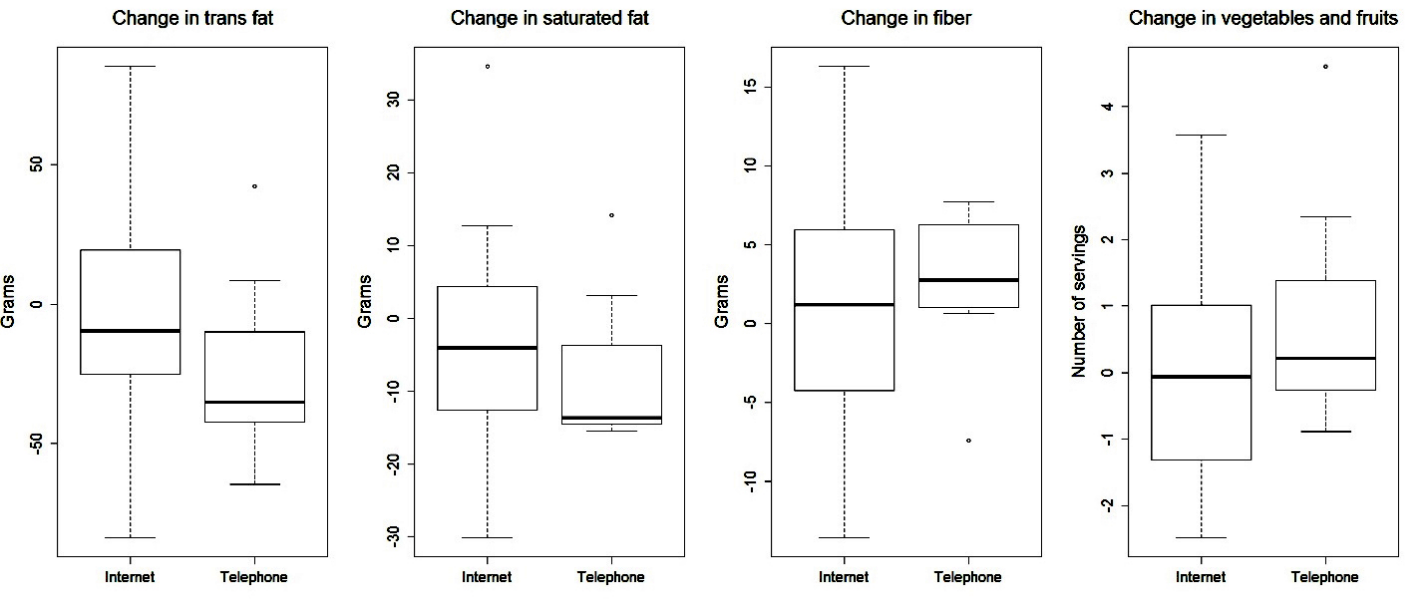
Boxplots for change in nutrition outcomes scores by treatment from baseline to 6-month follow-up.

**Figure 4:**
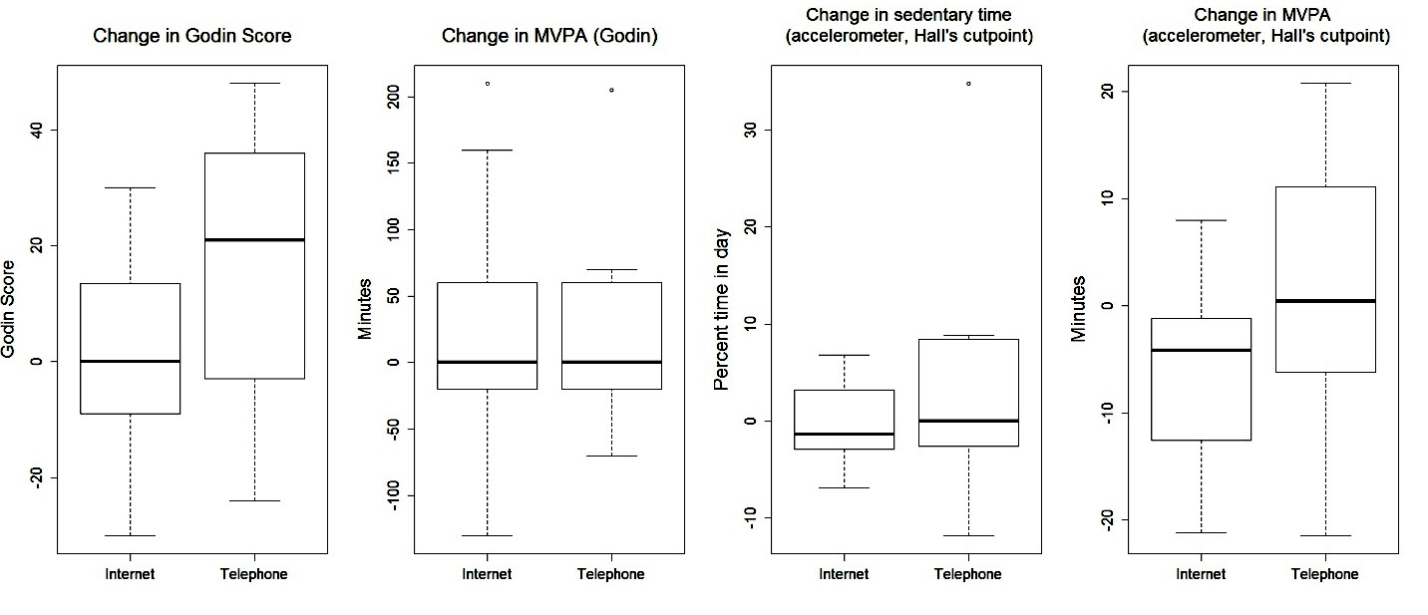
Boxplots of change in physical activity outcomes by treatment group from baseline to 6-month follow-up.

**Figure 5:**
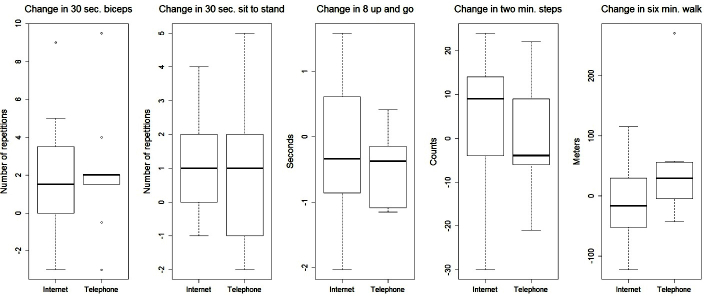
Boxplots of change in physical functioning outcomes by treatment group from baseline to 6-month follow-up.

Overall, between-group differences over time were only statistically significant for baseline to 6-month changes in waist circumference in favor of the telephone intervention (*P*=.03). Several other outcomes are worth noting including baseline to 6-month change in weight (*P*=.06), total body fat percentage (*P*=.09), body mass index (*P*=.08), and amount of fruit consumed (*P*=.10), all in favor of the telephone intervention. Figures 2 to 5 provide graphic depictions of these results. It is also worth noting that Figures 4 and 5 show potential differences between the intervention groups in terms of average number of sedentary minutes per day and the 6-minute walk test, respectively, with the telephone group having more sedentary minutes.

**Table 1:** Demographic information by treatment group.

Characteristic		Group	
		Telephone Mean (SD) or n (%) n=13	Internet Mean (SD) or n (%) n=24
Age, years, mean (SD)		59.92 (10.94)	59.62 (9.65)
**Sex, n (%)**			
	Male	2 (15)	5 (21)
**Type of cancer, n (%)**			
	Prostate	2 (15)	4 (17)
	Colon	0 (0)	2 (8)
	Endometrial	3 (23)	4 (17)
	Breast	8 (62)	14 (58)
**Ethnic background, n (%)**			
	Hispanic/Latino	3 (2.3)	3 (13)
**Race, n (%)**			
	Asian	0 (0)	1 (4)
	Black	3 (23)	3 (13)
	White	8 (61)	20 (83)
	Other	2 (15)	0 (0)
**Level of education, n (%)**			
	At least bachelor’s degree	7 (54)	11 (46)
	Less than bachelor’s degree	6 (46)	13 (54)
**Employment status, n (%)**			
	Employed full-time	6 (46)	11 (46)
	Not employed full-time	7 (54)	13 (54)
**Belong to religious group, n (%)**			
	Yes	13 (100)	18 (75)
**Present marital status, n (%)**			
	Married	8 (62)	14 (58)
	Not currently married	5 (39)	10 (42)
**Children^a^, n (%)**			
	At least one child	9 (75)	20 (83)
**Surgery, n (%)**			
	Yes	13 (100)	21 (88)
**Chemotherapy, n (%)**			
	Yes	6 (46)	10 (42)
**Radiation therapy, n (%)**			
	Yes	8 (62)	14 (58)
**Hormonal therapy^a^, n (%)**			
	Yes	7 (58)	11 (46)

^a^One person did not respond.

**Table 2:** Within-group baseline and 6-month follow-up means and standard deviations for measures of body composition, diet, physical functioning, and physical activity (note: mean and standard deviations were calculated for individuals who had observations for both baseline and follow-up).

	Intervention group	Baseline Mean (SD)	Follow-up Mean (SD)	Cohen *d*	*P* value
**Weight (kg)**					
	Telephone	82.07 (14.04)	77.53 (12.83)	0.81	.04
	Internet	86.62 (19.35)	86.28 (19.96)	0.12	.64
**Waist circumference (cm)**					
	Telephone	97.23 (8.81)	92.36 (10.7)	1.01	.02
	Internet	94.13 (11.98)	93.53 (11.45)	0.28	.30
**Total body fat (%)**					
	Telephone	41.49 (4.23)	40.23 (6.06)	0.41	.25
	Internet	43.33 (7.56)	44.06 (7.26)	0.98	.004
**Body mass index (kg/m^2^)**					
	Telephone	31.56 (3.07)	30.02 (4.06)	0.75	.06
	Internet	32.38 (5.05)	32.27 (5.49)	0.11	.68
**ASA24^a^trans fat (g/day)**					
	Telephone	66.61 (13.75)	42.05 (28.38)	0.72	.08
	Internet	60.86 (35.1)	58.69 (39.75)	0.05	.87
**ASA24 saturated fat (g/day)**					
	Telephone	20.41 (4.24)	12.27 (8.78)	0.75	.07
	Internet	21.17 (13.16)	18.08 (13.26)	0.19	.50
**ASA24 fiber (g/day)**					
	Telephone	15.15 (4.37)	17.7 (4.99)	0.53	.17
	Internet	14.39 (4.92)	15.15 (9.58)	0.08	.76
**ASA24 vegetables (servings/day)**					
	Telephone	1.58 (0.57)	1.9 (0.82)	0.31	.42
	Internet	1.39 (0.84)	1.44 (1.27)	0.03	.92
**ASA24 fruits (servings/day)**					
	Telephone	0.88 (0.73)	1.35 (0.99)	0.53	.18
	Internet	1.38 (1.13)	1.19 (1.02)	0.23	.41
**ASA24 vegetables and fruits (servings/day)**					
	Telephone	2.45 (0.82)	3.25 (1.58)	0.44	.25
	Internet	2.77 (1.33)	2.63 (1.86)	0.09	.74
**Godin physical activity score**					
	Telephone	33.33 (25.74)	48.33 (19.75)	0.56	.13
	Internet	23.6 (22.56)	25 (17.77)	0.08	.77
**Godin minutes of moderate or greater activity**					
	Telephone	129.44 (77.48)	156.67 (74.33)	0.34	.33
	Internet	66.88 (84.44)	88.75 (83.66)	0.27	.30
**30-second bicep curl, repetitions (2 arms average)**					
	Telephone	14.83 (3.81)	16.94 (2.96)	0.62	.10
	Internet	15.21 (3.25)	17.21 (3.46)	0.71	.02
**30-second sit-to-stand (repetitions)**					
	Telephone	12.22 (1.99)	13.33 (1.87)	0.5	.17
	Internet	11.36 (1.6)	12.29 (1.2)	0.73	.02
**8-foot up-and-go (seconds)**					
	Telephone	6.65 (1.28)	6.18 (0.99)	0.84	.04
	Internet	6.23 (1.07)	6.08 (1.23)	0.15	.60
**2-minute steps (count)**					
	Telephone	95.67 (16.96)	95.44 (16.61)	0.02	.96
	Internet	90.23 (22.13)	93.77 (16.66)	0.23	.42
**6-minute walk (meters)**					
	Telephone	476.36 (130.96)	519.06 (82.12)	0.46	.20
	Internet	490.18 (69.75)	478.55 (75.28)	0.18	.52
**Sedentary activity (minutes/day)**					
	Telephone	60.61 (10.09)	64.95 (15.96)	0.31	.41
	Internet	68.98 (5.69)	68.71 (4.92)	0.06	.84
**Moderate-to-vigorous activity (minutes/day)**					
	Telephone	36.22 (31.9)	37.49 (28.65)	0.09	.80
	Internet	16.17 (11.68)	10.6 (7.95)	0.63	.07

^a^ASA24: Automated self-administered 24-hour dietary recall.

## Discussion

### Principal Findings

This pilot RCT provides some of the first data directly comparing telephone- and Internet-delivered weight loss interventions among a sample of cancer survivors. Results suggest that the engagement was far greater with telephone intervention and consequently yielded larger improvements in several measures of body composition (especially waist circumference, which was highly significant), diet, physical activity, and physical fitness. Although outcomes generally favored the telephone group, participants with a higher percentage of body fat were more likely to drop out of this intervention group, indicating that the Internet intervention may be more acceptable for people with a high percentage of body fat. Previous research is mixed with regard to why participants drop out of weight loss interventions, but some research has found that for in-person interventions, weight or shape concerns may increase the likelihood of attrition [34].

Although our modest sample size and lack of statistical power hampered our ability to detect significant differences, several differences are worth noting as the effect sizes are clinically meaningful, including the percentage of body fat, fruits and vegetables consumed, moderate/vigorous physical activity (measured via accelerometer), and 6-minute walk test (found in Figures 2,4, and 5, respectively). Changes in these variables for participants in the telephone group were in the hypothesized direction, while participants in the Internet group showed changes in the opposite direction. In a larger sample, these differences may have been more pronounced. These findings are consistent with a recent review of telephone- and Web-based weight management interventions for cancer survivors which suggests telephone interventions may be more effective than Web-based approaches [15].

In terms of within-group change, the telephone group had more outcomes related to fitness and weight loss that changed over the 6 months than the Internet group. These included weight, waist circumference, and 8-foot up-and-go time. In terms of change in weight, participants in the telephone group experienced a 5.6% weight loss which is clinically meaningful [35]. Participants in this group also experienced a decrease of 5 cm in waist circumference, decreasing from 97.2 cm to 92.4 cm. Epidemiological research suggests an increased risk of all-cause mortality among individuals whose waist girth falls within the range of 95 to 100 cm as compared to those whose waists measure 90 to 95 cm (especially among women) [36]. Interestingly, the Internet group did have 2 measures of physical functioning change including the 30-second bicep curl and sit-to-stand, which suggest some advancements in strength training. This group also showed a significant increase in percentage of body fat; however, results indicating changes in physical functioning may be an artifact of multiple comparisons as there were no meaningful differences between interventions other than modality.

By examining the engagement data we can indirectly assess participant perceptions of usefulness or enjoyment. Engagement was assessed in the telephone group via the number of phone sessions out of 9 that they completed. In the Internet group, it was assessed as the number of days that they logged on during the intervention out of a possible 160 days. This finding is consistent with the reviews and meta-analyses of the previous literature [17,18,37], which suggest that more personal contact with participants leads to better improvements in outcomes. Moreover, these reviews reported that interventions with at least one in-person interaction resulted in greater engagement and better outcomes. In a recent systematic review of weight loss intervention for cancer survivors [38], the authors note that interventions that combined technology-based modalities (such as telephone) with in-person counseling were more effective than those using only one modality.

Engagement in Internet-delivered interventions is an ongoing area of research. One recent study found that an Internet-delivered intervention for cancer-related distress among survivors suggests that engagement tends to be higher for women, for participants who underwent chemotherapy, and when participants are recruited online [39]. The authors also note that the social networking component increased overall engagement but may have interfered with other intervention components. In a separate weight loss study using a Web-based intervention, researchers found no difference between an information-based website and 2 supportive ones—one that provided feedback, social support, and self-monitoring and another that provided the same features plus personalized planning [40]. Despite the lack of significant differences between websites in terms of weight loss, use of the supportive websites was higher compared to the informative website, suggesting that greater engagement may not lead to greater weight loss. It should be noted that completing 9 telephone counseling sessions may not be equivalent to logging on to the website every day. Moreover, we did not have any measure of the pattern of log-ins over time or what the participant did while they were logged on, limiting our ability to infer how much of the intervention material to which they were exposed.

We compared percent completion of online surveys across the 2 groups, and while differences were not statistically significant, studies with larger sample sizes may find significant differences. In order to address this issue, standardized measures of engagement should be developed to compare across Web-based and non–Web-based interventions. One possible measure may be length of time exposed to intervention materials (eg, length of telephone sessions in minutes and number of minutes participants spent logged in to the website). Researchers should continue to evaluate different measures in order to identify the most accurate measure of adherence.

### Strengths and Limitations

This study has several strengths worth noting. First, it is one of the first studies to directly compare a telephone-delivered intervention and an Internet-delivered intervention in a sample of cancer survivors. Second, several objective measures of body composition, physical activity, and physical performance were used to capture changes in important markers of health that may have occurred during the intervention. Last, the intervention that was delivered is easily disseminable and requires fewer resources compared to interventions that use supervised exercise sessions.

Although this pilot study had many strengths, there also were weaknesses. First is the small sample size. Few of the changes from pre- to postintervention were statistically significant; however, many of the between-group differences in outcomes, while not statististically significant in this study, would have likely been significant had the sample size been larger. Additionally, attrition for this study was fairly high with about 35% of participants dropping out before completing the study. Given the issues we encountered with attrition, future studies examining Web-based interventions for weight loss should account for potentially high attrition rates in the Web-based group by having proportionally more participants for this condition relative to other conditions. In the telephone group, participants with a higher percentage of body fat were more likely to drop out, and because of the small sample size, we were unable to control for this in our analyses. Finally, because many outcome variables were measured, many statistical comparisons were used, which could have increased the type I error rate; however, given that (1) this was a pilot study, (2) the goal of the pilot study was to identify relationships for future study, and (3) all comparisons were planned before the intervention was delivered, we felt that it was unnecessary to adjust for multiple comparisons.

### Conclusion

The results of this pilot study are compelling and provide direction for future studies. Specifically, future studies that compare telephone- and Internet-delivered interventions would benefit from techniques to enhance adherence and examine cost differences. It may prove beneficial to augment current weight loss interventions in health care settings with personalized intervention components. Research suggests that interest in technology-based interventions is influenced by the survivors’ current technology use, their age, and their current lifestyle patterns (eg, eating and physical activity behaviors) [41]. In fact, a program using both telephone and Web components may be able to maximize reach and engagement. Future studies should also focus on how to get older participants to engage more with technology so as to enhance Internet-based interventions. Over time, as younger participants who are more comfortable with technology age, there may be a shift in preference of intervention modality toward Internet-based or other technology-based interventions. A recent study involving breast cancer survivors showed moderate improvements in weight (2% weight loss), fruits/vegetable consumption (+1.5 servings/day), and physical activity (+5.75 metabolic equivalent of task hours per week) in an intervention using a multimodal mHealth approach [42]. Several outcome measures showed promise in terms of 6-month change including percentage of body fat, waist circumference, fruits and vegetables consumed, moderate/vigorous physical activity (measured via self-report), and 6-minute walk test. Future studies should focus on these outcomes.

Finally, studies should also determine the most effective intervention components and how to best combine these in order to create the most robust intervention strategy. As Hoedjes [38] notes, several promising theoretical components such as goal setting, action planning and social support may be effective for weight loss interventions for cancer survivors; however, optimizing the modality for delivery may be just as important. Future studies should use the multiphase optimization strategy to determine the most effective components [43].

## Appendix

Multimedia Appendix 1 Differences in baseline assessments between drop-outs and non–drop-outs by intervention group.

Multimedia Appendix 2 CONSORT Checklist.

## Abbreviations

ASA24: automated self-administered 24-hour dietary recall
BMI: body mass index
CONSORT: Consolidated Standards of Reporting Trials
RCT: randomized controlled trial
RENEW: Reach Out to Enhance Wellness
VO2max: maximal oxygen consumption
