# Supplementary material for: Comparison of Internet and Telephone Interventions for Weight Loss Among Cancer Survivors: Randomized Controlled Trial and Feasibility Study
Source: JMIR Cancer. 2017 Sep 27;3(2):e16. doi: 10.2196/cancer.7166 (PMC5637068; doi:10.2196/cancer.7166)
Supplement: Multimedia Appendix 1 [file cancer_v3i2e16_app1.pdf]

Multimedia Appendix 1. Differences in baseline assessments between drop-outs and non drop-outs by intervention group.

Missing Measure	Group	Drop-outs Mean (SD)	Non Drop-outs Mean (SD)	T	P-value
Weight	Telephone	117.10 (36.24)	82.07 (14.04)	-1.87	0.147
	Internet	90.02 (11.86)	86.62 (19.35)	-0.47	0.640
Waist	Telephone	112.90 (23.96)	97.23 (8.81)	-1.27	0.285
	Internet	100.60 (9.48)	94.13 (11.98)	-1.38	0.183
Days Since Diagnosis	Telephone	1768.80 (1589.90)	1880.00 (887.10)	0.16	0.872
	Internet	1762.10 (976.00)	1967.90 (1032.00)	0.48	0.634
DEXA Body Fat %	Telephone	51.85 (3.81)	41.49 (4.23)	-4.18	0.002
	Internet	41.95 (8.31)	43.33 (7.56)	0.43	0.675
ASA Total Fat	Telephone	92.01 (37.14)	66.61 (13.75)	-1.32	0.267
	Internet	81.20 (43.82)	60.86 (35.10)	-1.23	0.232
Godin PA Score	Telephone	12.00 (15.87)	33.33 (25.74)	1.33	0.214
	Internet	37.50 (23.57)	23.60 (22.56)	-1.39	0.180
30 Sec. Biceps Rep. (Two Arms Average)	Telephone	11.75 (3.18)	14.83 (3.81)	1.41	0.187
	Internet	13.38 (2.93)	15.08 (3.52)	1.29	0.210
Actigraph Average ***Minutes*** of Sedentary Activity Per Day (Based on Freedson cut point)	Telephone	401.8 (75.87)	311.7 (120.9)	-1.18	0.267
	Internet	279.4 (149.1)	373.8 (100.0)	1.72	0.102
Actigraph Average ***Percent*** of Below Activity Level Per Day (Based on Hall cut point)	Telephone	70.30 (7.02)	60.62 (10.09)	-1.51	0.166
	Internet	69.00 (14.43)	68.98 (5.69)	< 0.01	0.997
Actigraph Average ***Percent*** of Moderate to Vigorous Activity Per Day (Based on Hall cut point)	Telephone	8.83 (3.53)	11.61 (5.38)	0.82	0.436
	Internet	8.93 (6.01)	7.76 (2.37)	-0.60	0.56

Note 1: Drop-out was defined as having complete observations at baseline and missing any observation for any sub-measure at month 6.

Note 2: For Godin measures, only Godin physical activity score was considered in calculating drop-outs.

Note 3: For Actigraph data, some individuals with no baseline data had Actigraph measurement for month 6 (1 participant in telephone group and 3 participants in internet group). These individuals were not counted as drop-outs.
